# Laryngology

**Published:** 1896-12

**Authors:** Barclay J. Baron


### LARYNGOLOGY.

Mayo Collier[2] says that the effects of **chronic nasal obstruction** are: (1) dryness of the mouth and tongue in the morning, from breathing through the mouth during sleep, and this due to enlargement of the erectile tissues on the lower turbinated bone, known as turbinal varix; (2) disturbed sleep, and in children nightmare; (3) night cough from the effect of cold, dry, and unfiltered air entering the larynx and trachea; (4) morning cough and expectoration from engorgement and congestion of the mucous membrane of the trachea and larynx, due to the dry-cupping of the parts during the abnormal and impeded respiration; (5) hoarseness and pharyngeal, laryngeal, and post-nasal catarrh; (6) enlarged, inflamed, and morbid conditions of the tonsils oral, pharyngeal, and lingual; (7) deafness, dry catarrh of the tympanum, suppuration of the middle ear; (8) polypus of the nose and naso-pharynx, anterior and posterior hypertrophies, spurs, ridges, and turbinal varices, due to the chronic state of enlargement and irritation to which the parts are subjected by the ever-present diminished nasal tension and want of ventilation; (9) affection of the accessory sinuses of the nose; (10) effects on distant parts, *e.g.* chest-walls, spine, lung, &c. He also draws attention to the correct way to test the nasal respiration; viz., by telling the patient to *inspire* through the suspected nostril, closing the other one and the mouth. If he can exist for a minute or two without opening his mouth, there is no material degree of nasal obstruction

Bryson Delavan reports a case[3] of **multiple papillomata affecting both vocal cords**, in which the use of instruments was not desirable, which was completely cured by using a spray of alcohol. The patient sprayed the larynx six times a day for over four months.

These cases of multiple and often sessile papillomata are very difficult to treat, as it is impossible either to snare them off or to tear them away with forceps. I have used the galvano-cautery, but this is not a safe instrument to apply to the interior

[1] *Am. J. M. Sc.*, 1895, cx. 519. [2] *J. Laryngol.*, 1896, x. 117.

[3] *Ibid.*, 146.

of the larynx. The alcohol treatment may help us in this difficulty, and we know what it will do for aural polypi.

Mr. Watson Cheyne states[1] that **cancer of the pharynx** commences in 60 per cent. of the cases on the surface of the tonsils, and, as it causes little trouble in its early stages, is very apt to be overlooked. He has tabulated all the published cases in three groups, according to the extent of the disease or the method of removal: *Group I.* Where the disease was removed from the mouth with or without splitting the cheek and with or without tracheotomy—where there was no wound in the neck communicating with the mouth. *Group II.* Where the internal wound communicated with a wound in the neck. *Group III.* Where the disease involved both the pharynx and larynx. Reference should be made to Mr. Cheyne's elaborate statistics, which show that the mortality varies greatly in the different groups. If tracheotomy is necessary, there is nothing gained by performing it some days before the principal operation for removal of the growth. The lymphatic area ought to be freely cleared out, and the internal jugular ligatured and resected if any glands are adherent to its sheath. The external carotid was tied in some of the operations, but there is then risk of secondary hemorrhage. Septic pneumonia is also a cause of death, which has to be reckoned with. Leaving the external wound fully open will, probably, lessen this.

A discussion on **intratracheal injections** in diseases of the respiratory tract took place at the March meeting of the New York Academy of Medicine.[2] Most of those who spoke on the matter considered astringents, stimulants, and antiseptics could be applied with advantage by means of a suitable syringe to the mucous surface of the trachea and bronchi, especially volatile oils in oily media. Relief of harassing cough, absorption of inflammatory swellings, and cleansing of ulcerated surfaces follow this method of medication.

Felix Semon[3] has given us the important points in connection with **malignant disease of the larynx.** It is always primary, never secondary or metastatic, or attacks the larynx by contiguity only; it is most commonly epithelioma, sarcoma being very rare in this situation. It is much more often seen in males than females, and most of the cases occur between the ages of forty and seventy years. In intrinsic disease the vocal cords are most frequently first affected, and the great symptom is hoarseness, which may persist for months, or even a year or more, without any other symptom being present. Pain may or may

[1] *The Objects and Limits of Operations for Cancer*, 1896.
[2] *J. Laryngol.*, 1896, x. 319. [3] *Clin. J.*, 1896, vii. 265.

not make its appearance even up to the time of death. Slight and repeated hemorrhage is very characteristic, but may be absent. The disease may commence as simple congestion, followed by swelling, or may assume at once the form of diffuse swelling. It may begin as a globular, sessile, nodulated mass, or look like simple papilloma or fibroma. One very important point to remember is the localisation of the growth, which, if benignant, tends to be in the anterior parts of the vocal cords, and, if malignant, on the posterior parts, the interarytenoid area, the epiglottis, or aryteno-epiglottidean folds. The appearance, too, is different in the two classes of growth: the malignant growth is whiter in colour and the individual projections of it are much more pointed than is the case in the innocent ones. The impairment of mobility of a vocal cord is not a necessary accompaniment of malignant growth, because in its early stages it may be superficial. The average duration of life in cancer of the larynx is between two and three years. The cases most favourable for operation are those in which there is a definite tumour of one cord. Where the disease is too advanced for thyrotomy, a part or half of the larynx must be extirpated, and the cases most suitable for operation are those in which the disease is situated on the front parts of the larynx. Early tracheotomy is the best palliative where operation is not permissible.

It is the *early diagnosis* of these cases that is so very desirable, and where we have to do with a growth in the posterior parts of the larynx, whether on the cord or behind it, we should most carefully look for impairment of mobility of the cord *when the patient is quietly breathing*. Those of us who are constantly dealing with laryngeal cases know how difficult it is to distinguish between an epithelioma and pachydermia laryngis at a certain stage of both diseases; and amongst other points of difference is this one of impairment of mobility, which must never be overlooked.

Holloway[1] writes in favour of using nitrous oxide gas, alone or mixed with a small quantity of ether vapour, as an **anæsthetic in operations on the throat** and nose. He quotes over 4,000 cases in which this plan has been followed without bad result. He directs attention especially to the fact that we can operate with the patient in the sitting position if we use this anæsthetic, which is, of course, impossible with chloroform; and he claims the following advantages for this position: (1) We can easily reflect light into the mouth by means of the frontal mirror, and so control the steps of the operation. (2) It is quite safe for the patient. (3) Hemorrhage can be well controlled, because we can see its source, and by bending the head forward the blood easily runs out of the mouth and does not tend to find its

[1] *Med. Mag.*, June, 1896; abstract in *J. Laryngol.*, 1896, xi. 128.

way into the larynx. If chloroform is used, "Krohne's improved regulating inhaler" is very valuable, as it enables one to measure the amount of the anæsthetic given with such accuracy. Deep narcosis not being necessary for the operation of tonsillotomy or removal of adenoid growth, the cough reflex should not be abolished. In the last twelve cases in which chloroform has been given by the author, the average amount of the anæsthetic used was 18.5 minims for every *five minutes* during which anæsthesia was maintained.

I have operated on about twenty cases of adenoid growth with nitrous oxide, or this gas and oxygen mixed, as the anæsthetic, and with the patients in the sitting position; and with one of these patients had more anxiety, from the fact of blood or growth falling into the larynx directly the operation was over and before I could bend the head over a basin, than I have had in hundreds of other cases operated on in which chloroform was employed, and in which I have never had any anxiety in this operation from blood finding its way into the air-passages; and I always operate with the patient turned well over on to the right side, with the head hanging over the edge of the bed or table. The blood flows out of mouth and nose quite freely and breathing is not at all obstructed.

BARCLAY J. BARON.

